# *Khoonmengia honbaensis*, a new genus and species of temperate bamboo (Poaceae, Bambusoideae) from central-southern Vietnam

**DOI:** 10.3897/phytokeys.138.39512

**Published:** 2020-01-10

**Authors:** Yi-Hua Tong, Xi-Rong Zheng, You Yuan Zhang, Qiao-Mei Qin, Jing-Bo Ni, Tien Chinh Vu, Nian-He Xia

**Affiliations:** 1 Key Laboratory of Plant Resources Conservation & Sustainable Utilization/ Key Laboratory of Digital Botanical Garden of Guangdong Province, South China Botanical Garden, Chinese Academy of Sciences, Guangzhou, 510650, China South China Botanical Garden, Chinese Academy of Sciences, Guangzhou Guangzhou China; 2 University of Chinese Academy of Sciences, Beijing, 100049, China Southeast Asia Biodiversity Research Institute, Chinese Academy of Sciences Nay Pyi Taw Myanmar; 3 Southeast Asia Biodiversity Research Institute, Chinese Academy of Sciences, Yezin, Nay Pyi Taw 05282, Myanmar Guangzhou Institute of Forestry and Landscape Architecture Guangzhou China; 4 Vietnam National Museum of Nature, Vietnam Academy of Science and Technology, Hanoi Vietnam Guangdong Eco-engineering Polytechnic Guangzhou China; 5 Graduate University of Science and Technology, Vietnam Academy of Science and Technology Hanoi, Vietnam Vietnam National Museum of Nature, Vietnam Academy of Science and Technology Hanoi Vietnam; 6 Department of Botany, Guangzhou Institute of Forestry and Landscape Architecture, Guangzhou 510540, China Graduate University of Science and Technology Hanoi Vietnam; 7 Guangdong Eco-engineering Polytechnic, Guangzhou 510520, China Southeast Asia Biodiversity Research Institute, Chinese Academy of Sciences Nay Pyi Taw China

**Keywords:** Arundinarieae, morphology, phylogeny, scrambling bamboos, synflorescence

## Abstract

A new genus of Arundinarieae, *Khoonmengia*, is established to accommodate a unique new bamboo species, *K. honbaensis*, from central-southern Vietnam. The morphological features, habitats and distribution of *Khoonmengia* and related genera, i.e. *Ampelocalamus* and *Hsuehochloa*, are compared. The characters of its scrambling habit, internodes with brownish green dots, conspicuous nodes swollen at one side, elliptic buds wholly sunken into culm, extravaginal branching pattern, mid-culm branch complement with one central dominant branch elongating to reiterate the culm accompanied by several lateral slender branches, swollen culm sheath base with a distinctive zone of transverse wrinkles, synflorescence composed of only one spikelet, single or several to many synflorescences arranged into a raceme or panicle terminal on leafy branches, purple anthers and nut-like caryopsis with hardened pericarp and loosely adherent lemma and palea distinguish *K. honbaensis* from morphologically similar taxa. In order to investigate the phylogenetic position of this unknown bamboo, molecular phylogenetic analyses based on the nuclear gene GBSSI were also conducted, and the results proved that *K. honbaensis* is definitely a member of Arundinarieae with an isolated position, which also indicated that this species could not be assigned to any of the already described genera and supported the establishment of the new genus.

## Introduction

Bamboos, including a single evolutionary radiation of 1,642 species in the grass family Poaceae, subfamily Bambusoideae, are important components in tropical to warm temperate forests (Vorontsova et al. 2016). Bambusoideae is classified into two tribes of woody bamboos (the tropical Bambuseae and the temperate Arundinarieae) and one tribe of herbaceous bamboos (the Olyreae) (Soreng et al. 2015). The Arundinarieae are the temperate woody bamboos, a diverse clade of 31 genera and ca. 546 species, with the center of diversity in East Asia (ca. 430 species), distributed primarily in forests of the northern temperate zone, but also in some high elevation tropical regions (BPG 2012; Clark et al. 2015). Arundinarieae is not only a taxonomically difficult group of bamboos, but also a troublesome one in molecular phylogenetics (Yang et al. 2013). Previous phylogenetic studies mainly based on plastid DNA divided Arundinarieae into twelve lineages, but the phylogenetic relationships among many clades were not well resolved (Zeng et al. 2010; Zhang et al. 2012; Ma et al. 2014; Attigala et al. 2016). Some analyses also revealed many inconsistencies between the plastid and the nuclear gene trees (Zhang et al. 2012; Yang et al. 2013). For example, *Ampelocalamus actinotrichus* had an affinity with individuals of *Chimonocalamus* in the plastid phylogeny (Zeng et al. 2010), rather than other taxa of *Ampelocalamus*. However, in the nuclear gene phylogenies, this species formed a clade with the congeneric taxa (Yang et al. 2013). These results implied that the nuclear genome and the plastid genome may have different evolutionary trajectories (Zhang et al. 2012; Yang et al. 2013). The most recent study based on phylogenetic analyses with RAD-seq data identified eight major lineages in Arundinarieae with strong support, which conflicts with earlier studies (Wang et al. 2017).

During an investigation of the bamboos in Hon Ba Nature Reserve, Khanh Hoa Province of central-southern Vietnam in October 2017, an unusual bamboo with unicaespitose habit, scandent stems, pachymorph rhizomes and semelauctant inflorescences on leafy flowering branches caught our attention. This species was misidentified as *Bambusa tulda* Roxb. during the background survey of Hon Ba Nature Reserve (Lee et al. 2014). The semelauctant inflorescence is a relatively rare condition in the Bambuseae. After closer examination, we found that it has three stamens and two stigmas. By its habit and floral characters, it should be a member of the tribe Arundinarieae. In Vietnam, only five clambering genera, i.e. *Melocalamus* Benth., *Maclurochloa* K. M. Wong, *Nianhochloa* H. N. Nguyen & V. T. Tran, *Cochinchinochloa* H. N. Nguyen & V. T. Tran and *Yersinochloa* H. N. Nguyen & V. T. Tran, are currently recognized, but all of these genera belong to Bambuseae (Wong 1993; Tran et al. 2013). In Southeast Asia, many other genera also have climbing or clambering culm habits, such as *Holttumochloa* K. M. Wong, *Kinabaluchloa* K. M. Wong, *Dinochloa* Büse, *Racemobambos* Holttum, *Chloothamnus* Büse, etc. However, none of these genera belong to Arundinarieae either. There are only two genera belonging to Arundinarieae in subtropical Asia, i.e. *Ampelocalamus* S. L. Chen, T. H. Wen & G. Y. Sheng and *Hsuehochloa* D. Z. Li & Y. X. Zhang, which have a combination of morphological characters including scandent stems, semelauctant inflorescences, three stamens and two stigmas (Chen et al. 1981; Zhang et al. 2018). However, this unknown bamboo has some both reproductive and vegetative characters that are different from these two subtropical genera (see Table 1).

**Table 1.** Comparison of morphological characters, distributions and habitats of *Khoonmengia*, *Hsuehochloa* and *Ampelocalamus*.

	*** Khoonmengia ***	*** Hsuehochloa ***	*** Ampelocalamus ***
Habit	Scrambling	Pendulous or procumbent	Scrambling
Branching pattern	Extravaginal	Extravaginal	Transferring
Nodes	Swollen at one side	Nearly flat	Swollen at one side
Mid-culm branch complement	One central dominant branch accompanied by 1–4 lateral slender ones	3–7 branches, subequal	Several to numerous branches, subequal, or one or three dominant branches accompanied by numerous slender ones
Bud	Elliptic, wholly sunken into culm	Elliptic, wholly sunken into culm	Ovate to broad ovate, not sunken or only base sunken into culm
Culm sheath base	Swollen, with a distinctive zone of transverse wrinkles	Flat, without a distinctive zone of transverse wrinkles	Usually swollen, without a distinctive zone of transverse wrinkles
Culm sheath auricles	Absent	Present, falcate, amplexicaul	Absent or present
Culm sheath oral setae	Absent	Present, radiate	Absent or present
Presence of dots on culm	With brownish green dots	Without dots	Without dots
Leaf auricles and oral setae	Absent	Present	Absent or present
Synflorescence	Composed of only one spikelet, single or several to many synflorescences arranged into a raceme or panicle on leafy flowering branches	Racemose, composed of 1 or few spikelets, single synflorescence on leafy flowering branches	Paniculate, composed of many spikelets, on leafy or leafless flowering branches
Glumes	(0–)1–2	Unknown	2
Number of florets per spikelet	7–9	5	2–7
Anther color	Purple	Purple	Yellow
Caryopsis	Nut-like, with hardened pericarp and loosely adherent lemma and palea	Unknown	Grain-like, without hardened pericarp and with closely adherent lemma and palea
Distribution	Central-southern Vietnam	Southwest China (Guizhou)	South and Southwest China (Gansu, Chongqing, Sichuan, Guizhou, Yunnan, Hainan)
Habitat	Granite montane, alt. 1500 m	Limestone montane, alt. 500–950 m	Limestone, granite or basalt montane, alt. 200–1800 m

The nuclear gene GBSSI (granule-bound starch synthase I) occurs as a single copy in Poaceae and was often used in recent phylogenetic studies on woody bamboos (Zhang et al. 2012; Goh et al. 2013; Yang et al. 2013). Zhang et al. (2012) showed that the phylogeny based on GBSSI was better resolved at the generic level than the plastid phylogeny. Therefore, in order to investigate the phylogenetic position of this unknown bamboo, we conducted molecular phylogenetic analyses of Asian Arundinarieae based on GBSSI.

## Materials and methods

### Sampling and morphological study

Samples of this putative new species were collected for morphological and molecular phylogenetic studies from the only known population in Hon Ba Nature Reserve, Khanh Hoa Province, central-southern Vietnam during our field investigation in Oct. 2017. Photographs were taken with a CANON EOS 60D camera and dried flowers were dissected and examined under an Olympus SZX16 Microscope; line drawings and descriptions were made by reference to dried specimens.

### DNA amplification and sequencing

Total genomic DNA was isolated from silica gel-dried leaf material using the Plant Genomic DNA Extraction Kit (Tiangen, Beijing, China), following the manufacturer’s instructions. The nuclear GBSSI sequence was amplified following the protocol used in Zeng et al. (2010). All PCR were performed in 25 μL volumes with a SensoQuest Labcycler 48 Gradient. A fragment from this unknown species was successfully sequenced by the DNA sequencing facility at Sangon Biotech (China). Automated sequencing output was checked visually for correct automated base-calling. Sequences were aligned using Bioedit v7.2.0 (Hall 1999) and adjusted manually where necessary. The newly obtained sequence has been deposited in Genbank.

In addition, sequences from the other 42 taxon representing nearly all known genera of Arundinarieae and outgroups, mainly following prior studies (Zeng et al. 2010; Zhang et al. 2012; Yang et al. 2013; Attigala et al. 2016), were downloaded from the NCBI Genbank database (http://www.ncbi.nlm.nih.gov/genbank/) (Table 2). *Bonia amplexicaulis* (L. C. Chia, H. L. Fung & Y. L. Yang) N. H. Xia, *Neomicrocalamus prainii* (Gamble) Keng f., and *Bambusa ventricosa* McClure of the tribe Bambuseae were chosen as outgroups based on prior studies (Zeng et al. 2010; Zhang et al. 2012).

**Table 2.** Voucher information and GenBank accession numbers for taxa used in this study.

Taxon	Voucher no.	Source	GenBank accession no. (GBSSI)
*Acidosasa chinensis* C. D. Chu & C. S. Chao ex Keng. f.	Zhang 08035 (KUN)	Guangdong, China	JN132035
*Acidosasa chienouensis* (T. H. Wen) C. S. Chao & T. H. Wen	Zhang 08065 (KUN)	Fujian, China	JN132043
*Ampelocalamus actinotrichus* (Merr. & Chun) S. L. Chen, T. H. Wen & G. Y. Sheng	Zeng and Zhang 06054 (KUN)	Hainan, China	KM264660
*Arundinaria gigantea* (Walter) Muhl.	Zhang US1025 (KUN)	Arkansas, United States	JN131985
*Arundinaria tecta* (Walter) Muhl.	Triplett 173 (ISC)	South Carolina, United States	JN131988
*Bambusa ventricosa* McClure	Zhang KMBG09 (KUN)	Yunnan, China	JN131925
*Bashania abietina* T. P. Yi & L. Yang	Zhang 07092 (KUN)	Sichuan, China	JN132004
*Bonia amplexicaulis* (L. C. Chia, H. L. Fung & Y. L. Yang) N. H. Xia	Zeng and Zhang SB5 (KUN)	Yunnan, China	JN131926
*Brachystachyum densiflorum* (Rendle) Keng	Zeng and Zhang 06174 (KUN)	Zhejiang, China	JN131957
*Chimonobambusa macrophylla* (Hsueh & T. P. Yi) T. H. Wen & Ohrnb	Zhang 07091 (KUN)	Sichuan, China	JN131980
*Chimonocalamus montanus* Hsueh & T. P. Yi	Zhang 07057 (KUN)	Yunnan, China	JN132029
*Chimonocalamus pallens* Hsueh & T. P. Yi	Zhang 07071 (KUN)	Yunnan, China	JN132060
*Drepanostachyum ampullare* (T. P. Yi) Demoly	GLM 081860 (KUN)	Xizang, China	JN132079
*Drepanostachyum hookerianum* (Munro) Keng f.	DZL 199903 (KUN)	Kew, Britain	AF445165
*Fargesia decurvata* J. L. Lu	Zhang 07087 (KUN)	Hubei, China	JN131937
*Fargesia fungosa* T. P. Yi	Zhang 07048 (KUN)	Yunnan, China	JN131982
*Fargesia nitida* (Mitford) Keng f. & T. P. Yi	Zhang KMBG10 (KUN)	Sichuan, China	JN131941
*Ferrocalamus strictus* Hsueh & Keng f.	Zeng and Zhang SB1 (KUN)	Yunnan, China	JN132090
*Gaoligongshania megalothyrsa* (Hand.-Mazz.) D. Z. Li, Hsueh & N. H. Xia	JRX 9401 (KUN)	Yunnan, China	JN131945
*Gelidocalamus rutilans* T. H. Wen	Zeng and Zhang 06183 (KUN)	Zhejiang, China	JN131967
*Himalayacalamus falconeri* (Munro) Keng f.	GLM 081524 (KUN)	Xizang, China	JN132078
*Hsuehochloa calcarea* (C. D. Chu & C. S. Chao) D. Z. Li & Y. X. Zhang	Zhen-Hua Guo 013 (KUN)	GenBank	KM264662
*Indocalamus sinicus* (Hance) Nakai	Zeng and Zhang 06081 (KUN)	GenBank	JN131939
*Indocalamus wilsonii* (Rendel) C. S. Chao & C. D. Chu	Zeng and SD Zhang 07119 (KUN)	GenBank	JN131928
*Indosasa crassiflora* McClure	Zhang 07014 (KUN)	GenBank	JN132069
*Khoonmengia honbaensis* N. H. Xia, Y. H. Tong & X. R. Zheng	BVN2017048 (IBSC)	Vietnam	MN521458
*Neomicrocalamus prainii* (Gamble) Keng f.	LL07236 (KUN)	Xizang, China	JN131921
*Oldeania alpina* (K. Schum.) Stapleton	Triplett and Clark (2010), ZHZ200101 (KUN)	Locality unkown	AF445171
*Oligostachyum sulcatum* Z. P. Wang & G. H. Ye	Zhang 07024 (KUN)	Guangxi, China	JN131987
*Ampelocalamus loudianensis* T. P. Yi & R. S. Wang	MPF10052 (KUN)	Guizhou, China	KM264663
*Ampelocalamus melicoideus* Keng f.	MPF10142 (KUN)	Chongqing, China	KM264667
*Ampelocalamus microphyllus* Hsueh & T. P. Yi	MPF10123 (KUN)	Chongqing, China	KM264665
*Ampelocalamus patellaris* (Gamble) Stapleton	Zhang 07075 (KUN)	Yunnan, China	AF445163
*Ampelocalamus scandens* Hsueh & W. D. Li	Zhen-Hua Guo 013 (KUN)	Yunnan, China	AF445164
*Phyllostachys edulis* (Carriere) Houzeau	Zhang KMBG04 (KUN)	Yunnan, China	JN132018
*Pleioblastus gramineus* (Bean) Nakai	Zhang and Zeng 06157 (KUN)	Zhejiang, China	JN131990
*Pleioblastus juxianensis* T. H. Wen, C. Y. Yao & S. Y. Chen	Zhang and Zeng 06136 (KUN)	Zhejiang, China	JN132037
*Pseudosasa japonica* (Sieb. & Zucc.) Makino	Zhang 07023 (KUN)	Guangxi, China	JN132010
*Sasa senanensis* Rehder	Triplett 146 (KUN)	Tennessee, United States	JN132068
*Sinobambusa tootsik* (Sieb.) Makino	Zhang and Zeng 06090 (KUN)	Guangdong, China	JN132015
*Thamnocalamus spathiflorus* (Trin.) Munro	GLM 081775 (KUN)	Xizang, China	JN132083
*Yushania basihirsuta* (McClure) Z. P. Wang & G. H. Ye	Zeng and Zhang 06108 (KUN)	Hunan, China	JN131961
*Yushania brevipaniculata* (Hand.-Mazz.) T. P. Yi	Zhang 08005 (KUN)	Sichuan, China	JN131933

### Phylogenetic analyses

Gaps were coded as present or absent using the simple indel coding method (Simmons and Ochoterena 2000). The best-fitting models were selected using jModeltest v2.1.4 under the Akaike Information Criterion (AIC) (Darriba et al. 2012). The model used for the GBSSI in this study was TrNef+G.

Phylogenetic analyses were conducted with PAUP*v.4.0b10, MrBayes 3.2.5 (Ronquist et al. 2012) and GARLI 2.0 (Zwickl 2006). MP analyses were conducted using PAUP*v.4.0b10 (Swofford 2003). Heuristic searches were performed with 1000 homogeneity replicates, tree-bisection-reconnection (TBR) branch swapping, MULTREES option off, and random addition of sequences with 1000 replicates.

ML analyses were conducted using GARLI 2.0 (Zwickl 2006), with 1000 bootstrap replicates. 50% majority-rule consensus tree was constructed using PAUP*4.0b10.

BI analyses were conducted using MrBayes 3.2.5 (Ronquist et al. 2012). The runs were conducted starting with random trees, consisting of a single cold chain and three heated chains, with the temperature set to 0.1. The Markov chain Monte Carlo (MCMC) chains were run for 10 million generations and sampled trees every 1000 generations for the GBSSI gene data set. A 50% majority-rule consensus tree was constructed from the remaining trees, yielding the posterior probability (PP) values for each clade.

## Results

**Figure 1.**
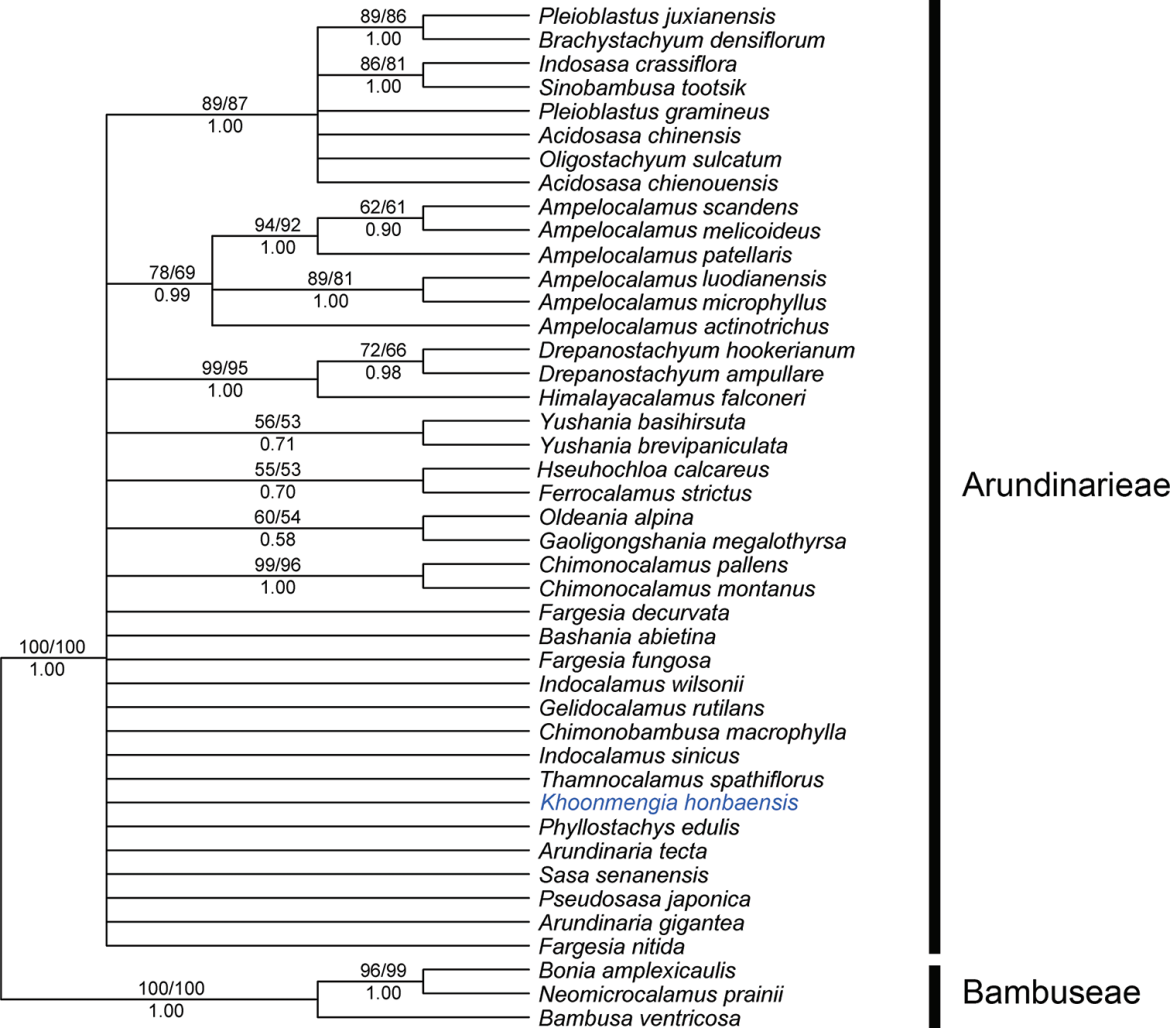
The strict consensus of 234 equally most parsimonious trees based on the partial GBSSI gene. Numbers below branches indicate posterior probability of Bayesian Analysis, and numbers above branches indicate bootstrap values of MP and ML.

A total of 1414 characters were included in the maximum parsimony (MP) analyses matrix, of which 133 characters were parsimony-informative, 238 variable characters were parsimony-uninformative and 843 characters were constant. The strict consensus tree for the 234 most parsimonious trees (tree length = 531; CI = 0.787; RI = 0.675; RC = 0.532) is shown in Fig. 1. The results of the MP, BI and ML analyses were almost identical except for slight position changes of some species (not shown). PP (posterior probabilities), MPBS (maximum parsimony bootstrap support) and MLBS (maximum likelihood bootstrap support) were included on the strict consensus tree from MP analyses. PP< 0.95 and MPBS/MLBS < 70% were considered as lacking support for a clade.

In the current study, the monophyly of the temperate woody bamboo clade was strongly supported, with 100% MPBS, 100% MLBS and 1.00 PP. Our putative new species was nested in the monophyletic clade of Arundinarieae. However, the phylogenetic relationships among groups of temperate woody bamboos were not resolved in this study.

## Taxonomic treatment

### Khoonmengia

Taxon classificationPlantaePoalesPoaceae

N.H.Xia, Y.H.Tong & X.R.Zheng gen. nov.

4FF08FA3-3412-5FB7-8C08-B08CD5A138C9

urn:lsid:ipni.org:names:77204206-1

#### Type.

*Khoonmengia honbaensis* N.H.Xia, Y.H.Tong & X.R.Zheng.

#### Diagnosis.

*Khoonmengia* resembles *Hsuehochloa* and *Ampelocalamus* in having pachymorph and short-necked rhizomes, florets with 3 stamens and 2 stigmas, but differs from the former by its scrambling habit, nodes swollen at one side, mid-culm branch complement with one central dominant branch elongating to reiterate the culm accompanied by 1–4 lateral slender branches, swollen culm sheath base with a distinctive zone of transverse wrinkles, culm and leaf sheaths without auricle or oral setae, and single or several to many synflorescences arranged into a raceme or panicle, and can be distinguished from the latter by extravaginal branching pattern, buds wholly sunken into culm, culm sheath base with a distinctive zone of transverse wrinkles, synflorescence composed of only one spikelet, purple anthers and nut-like caryopsis with hardened pericarp and loosely adherent lemma and palea.

#### Description.

Shrubby bamboo. Rhizomes pachymorph, short-necked. Culms unicaespitose, erect at lower part, distally scrambling; internodes terete, with dense brownish green dots; nodes conspicuous, swollen at one side. Buds elliptic, wholly sunken into culm. Branches extravaginal, often solitary at lower part of culm, and usually with one central dominant branch elongating to reiterate the culm and 1–4 lateral slender ones in the middle part of culm. Culm sheaths persistent, basally swollen, with a distinctive zone of transverse wrinkles; auricles and oral setae absent; blade reflexed; ligule convex. Foliage leaves without auricles and oral setae; ligules convex. Synflorescence semelauctant, composed of only one spikelet subtended by one or several sheath-like bracts, single or several to many synflorescences arranged into a raceme or panicle which is terminal on leafy branches; spikelets with 8–9 florets. Glumes (0-)1–2. Palea slightly shorter than lemma. Lodicules 3. Stamens 3, filaments free, anthers purple. Styles 2, free, stigmas 2, plumose. Caryopsis nut-like, with a hardened pericarp and loosely adherent lemma and palea, apex with 2 persistent style bases.

### Khoonmengia honbaensis

Taxon classificationPlantaePoalesPoaceae

N.H.Xia, Y.H.Tong & X.R.Zheng sp. nov.

86D0F33B-1A93-5C61-8572-564248A75BB7

urn:lsid:ipni.org:names:77204207-1

#### Type.

Vietnam, Khanh Hoa, Hon Ba Nature Reserve, 1500 m, 17 October 2017, N. H. Xia et al. BVN2017048 (holotype, IBSC!; isotypes, SING!, VNM!).

#### Description.

Culms erect at lower part, distally scrambling, 2–4(-10) m long; internodes terete, 20–32 cm long, 4–6 mm in diam., initially light purple, becoming gray-green, with dense brownish green dots turning black when dry; nodes conspicuous, swollen at one side, lower margin ciliate, supranodal ridge inconspicuous, intranodes glabrous. Buds elliptic, wholly sunken into culm. Branches extravaginal, often solitary at lower part of culm, and usually with one central dominant branch elongating to reiterate the culm and 1–4 lateral slender ones in the middle part of culm, lateral branches 10–25 cm long. Culm sheaths persistent, leathery, glossy, initially light purple, 8–9.5 cm long, abaxially with distinct veins, basally swollen, with a distinctive zone of transverse wrinkles; auricles and oral setae absent; blade reflexed, lanceolate, 6–9 cm long, glabrous, deciduous; ligule convex, ca. 2 mm high, glabrous. Leaves 3–8 per ultimate branch; leaf sheaths glabrous; auricles and oral setae absent; ligules convex, ca. 2 mm high; blades elliptic-lanceolate, 10–20 × 1–2.5 cm, glabrous both sides except margin ciliate at the base when young, secondary veins 3–5 pairs, transverse veins distinct. Synflorescence semelauctant, composed of only one spikelet subtended by one or several sheath-like bracts, single or several to many synflorescences arranged into a raceme or panicle which is terminal on leafy branches; spikelets 4–7 cm long, florets 8–9. Glumes (0-)1–2, ovate, ca. 10 × 3 mm, apex acute, glabrous, 11-veined. Rachilla segments flat, ca. 6 mm, glabrous, apex inflated. Lemma ovate-lanceolate, 12–13 × 5 mm, glabrous, 13-veined, apex acute with a mucro; palea slightly shorter than lemma, 11–12 × 2–3 mm, 2-keeled, keels ciliolate, apex with excurrent keel vein, 3-veined between keels and 2-veined outside keels, veins inconspicuous; lodicules 3, ovate, membranous, 3–5 × 1.5–2 mm, ciliolate, apex acuminate; stamens 3, filaments white, free, anthers tinged purplish when young, then becoming purple, ca. 7 mm long; ovary ovoid, 1 mm long, glabrous; styles 2, free, ca. 1 mm long, stigmas plumose, ca. 3 mm long. Caryopsis nut-like, with a hardened pericarp and loosely adherent lemma and palea, dark brown, fusiform, 8–9 × ca. 3 mm, apex with 2 persistent style bases.

#### Etymology.

*Khoonmengia* is named in honor of Dr. Khoon Meng Wong, a renowned botanist who has studied the bamboos and other plant groups of Southeast Asia for more than 35 years. The specific epithet is named after Hon Ba Nature Reserve, the type locality of this species.

#### Distribution and habitat.

This species was only found in the type locality, i.e. Hon Ba Nature Reserve, Khanh Hoa Province of Vietnam. It occurs in high mountain broadleaved forests at an elevation of ca. 1500 m.

#### Additional specimen examined

(paratype): VIETNAM, Khanh Hoa, Hon Ba Nature Reserve, 12°06'39.2"N, 108°56'47.2"E, C. Y. Lee et al. HIKK370 (HN!).

## Discussion

**Figure 2.**
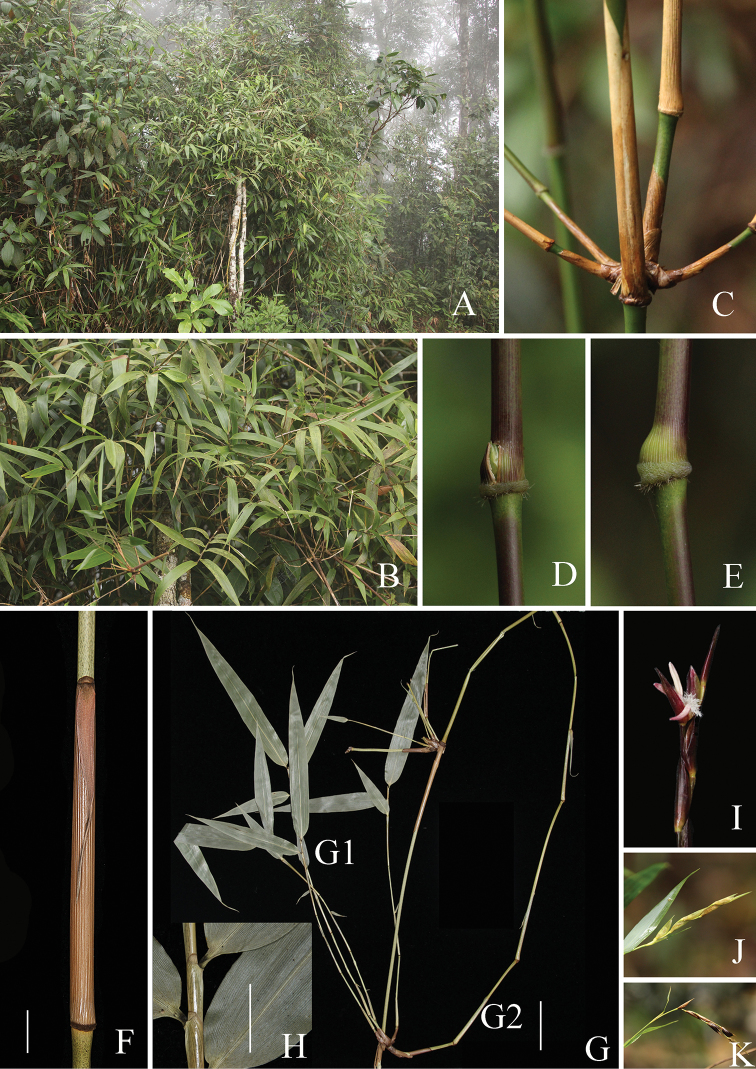
*Khoonmengia honbaensis***A** habit **B** leafy branches **C** branch complement **D** node with buds breaking out of the culm sheath base **E** node with bud inside the intact culm sheath **F** culm sheath **G** leafy branches at culm apex (G1 Slender branches, G2 Dominant branch) **H** leaf sheath **I** florets **J** synflorescence composed of only one spikelet **K** infructescence (**F, G, H** from N. H. Xia et al. BVN2017048). Scale bars: 1 cm (**F, H**); 5 cm (**G**).

**Figure 3.**
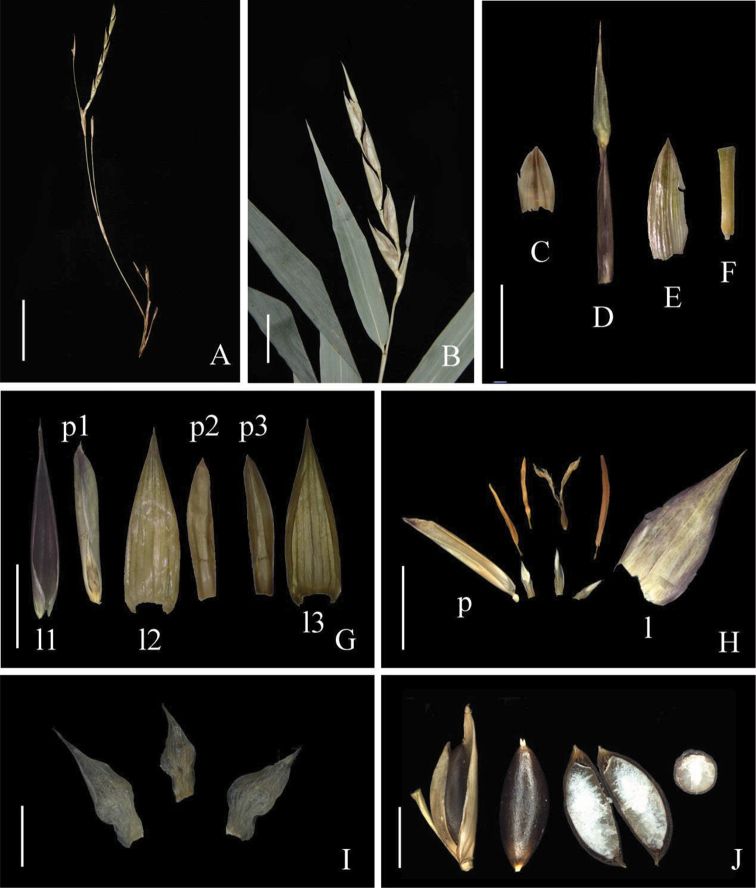
*Khoonmengia honbaensis***A** many synflorescences arranged into a panicle (leaf flowering branch not shown) **B** single synflorescence composed of only one spikelet subtended by a sheath-like bract terminal on leaf flowering branch **C** prophyll **D** sheath-like bract **E** glume **F** rachilla segment **G** lemmas & paleas **H** dissection of one floret showing lemma, palea, 3 stamens, gynoecium with 2 stigmas and 3 lodicules **I** lodicules **J** nut-like caryopsis (leftmost, within its lemma and palea, and second from left, detached) and when sectioned vertically (third from left) and transversely (rightmost). (l = lemma, p = palea). Scale bars: 2 cm (**A**); 1 cm (**B**); 5 mm (**C–H, J**); 2 mm (**I**).

**Figure 4.**
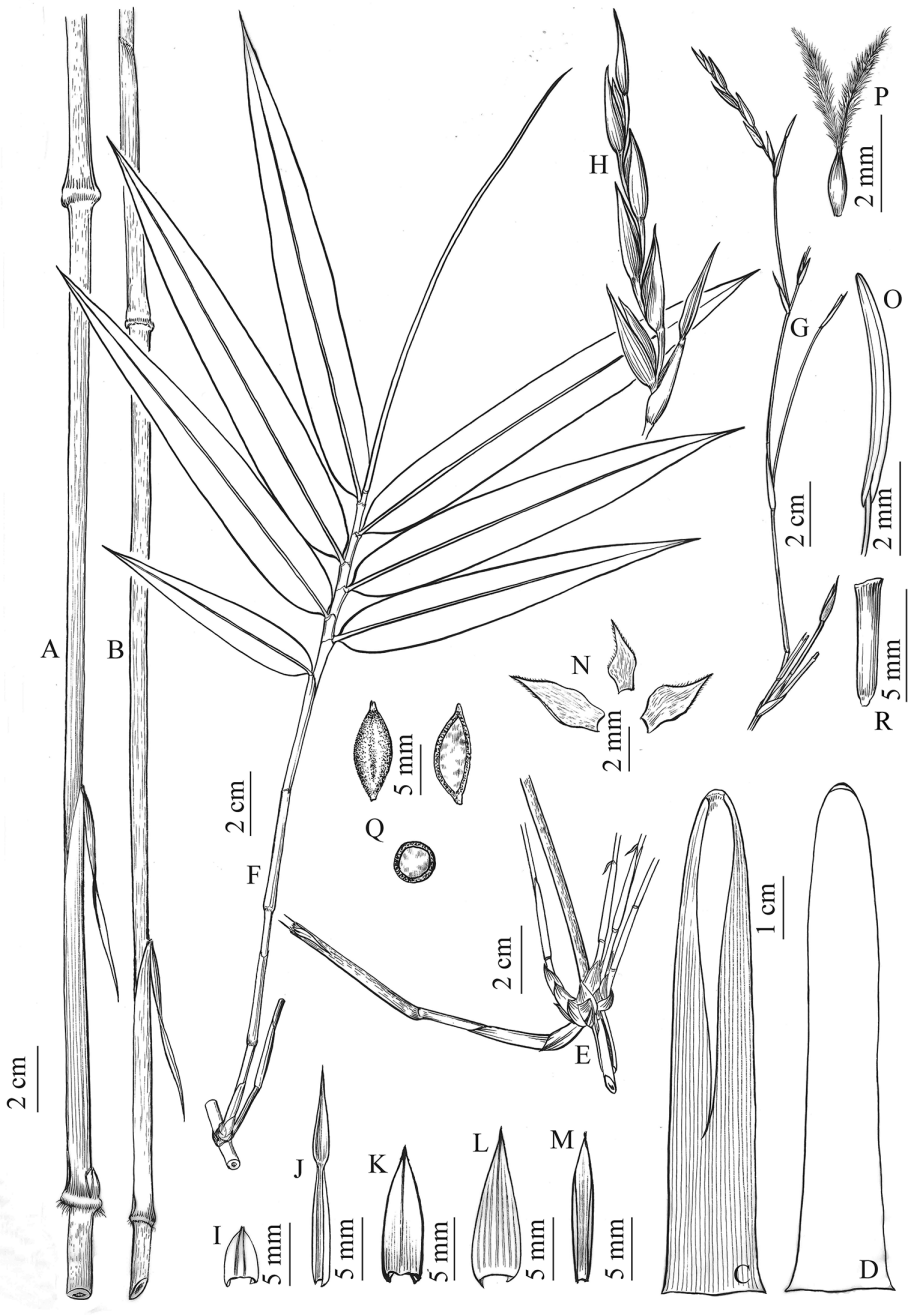
*Khoonmengia honbaensis***A, B** culm nodes and internodes with sheaths **C** culm sheath, abaxial view **D** culm sheath, adaxial view **E** branch complement **F** leafy branch **G** synflorescences arranged into a panicle **H** synflorescence composed of only one spikelet subtended by a sheath-like bract **I** prophyll **J** sheath-like bract **K** glume **L** lemma **M** palea **N** lodicules **O** stamen **P** pistil **Q** caryopsis and its vertical and cross sections **R** rachilla segment (From N. H. Xia et al. BVN2017048).

**Figure 5.**
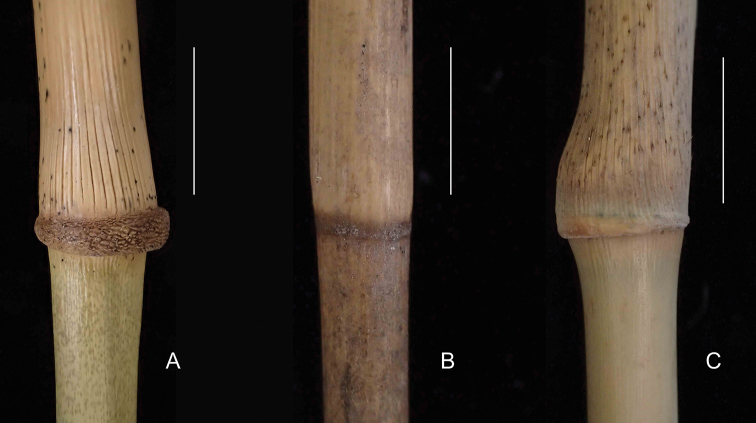
Comparison of culm leaf sheath bases **A***Khoonmengia honbaensis*, showing swollen culm leaf sheath base with a distinctive zone of transverse wrinkles (From N. H. Xia et al. BVN2017048) **B***Hsuehochloa calcarea*, showing flat and smooth culm leaf sheath base (From Y. Y. Zhang zyy-030, IBSC) **C***Ampelocalamus actinotrichus*, showing slightly swollen and nearly smooth culm leaf sheath base (From N. H. Xia et al. HN-025, IBSC). Scale bars: 1 cm (**A, C**); 5 mm (**B**).

**Figure 6.**
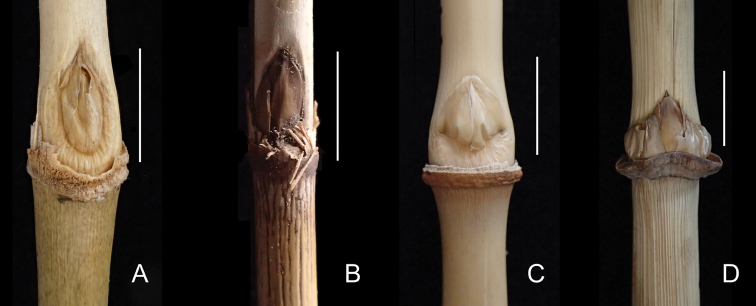
Comparison of buds **A***Khoonmengia honbaensis*, showing elliptic bud wholly sunken into culm (From N. H. Xia et al. BVN2017048) **B***Hsuehochloa calcarea*, showing elliptic bud wholly sunken into culm (From Y. Y. Zhang zyy-030, IBSC) **C***Ampelocalamus actinotrichus*, showing ovate bud with the base sunken into culm (From N. H. Xia et al. HN-025, IBSC) **D***Ampelocalamus melicoideus*, showing broad ovate bud not sunken into culm (From Y. Y. Zhang zyy-033, IBSC). Scale bars: 1 cm (**A, C, D**); 5 mm (**B**).

Morphological analysis (Table 1) revealed that this unknown bamboo owns several unique vegetative and reproductive characters that are different from the two closely related genera, i.e. *Ampelocalamus* and *Hsuehochloa*, such as culm with brownish green dots (Figs 2F, 5A), swollen culm sheath base with a distinctive zone of transverse wrinkles (Fig. 5A), synflorescence composed of solitary spikelet, single (Figs 2J–K, 3B, 4H) or several to many synflorescences arranged into a raceme or panicle (Figs 3A, 4G) terminal on leafy flowering branches, and nut-like caryopsis with loosely adherent lemma and palea (Fig. 3J). The nut-like bamboo caryopsis is different from the usual grain-like one by the hardened pericarp, and is reported only in some species of Bambuseae such as *Cephalostachyum pallidum* Munro, *Dendrocalamus membranaceus* Munro and *D. strictus* Nees before (Yu et al. 1993). Thus, the nut-like caryopsis type seems very rare in Arundinarieae. Our unknown bamboo species is also different from *Ampelocalamus* in the extravaginal branching pattern (vs. transferring), elliptic buds wholly sunken into culm (vs. ovate to broad ovate, not sunken or only base sunken into culm) (Fig. 6), and purple anthers (vs. yellow). For these three important generic characters, *Hsuehochloa* is the same as our unknown bamboo, which makes us infer that the closest genus to *Khoonmengia* may be *Hsuehochloa*. Although some important characters of *Hsuehochloa* such as number of glume, caryopsis type, are still unknown, besides the differences mentioned above, our unknown bamboo species can be further distinguished from *Hsuehochloa* by its scrambling habit (vs. pendulous or procumbent, not scrambling, according to the third author’s field observation), nodes swollen at one side (vs. flat, Fig. 5B), mid-culm branch complement with one central dominant branch elongating to reiterate the culm accompanied by 1–4 lateral slender branches (vs. subequal 3–7 branches), and culm and leaf sheath auricle or oral setae absent (vs. present). Moreover, *Hsuehochloa* grows on the limestone mountain, while the unknown species grows in granitic montane broadleaved forests. More detailed comparisons of these genera are summarized in Table 1. The GBSSI phylogeny revealed that our putative new species is definitely a member of Arundinarieae with an isolated position, which indicated that this species could not be assigned to any of the already described genera. Based on the above analysis of morphology, molecular phylogenetic relationships and habitat, we propose to establish a new genus to accommodate this unknown bamboo.

## Supplementary Material

XML Treatment for Khoonmengia

XML Treatment for Khoonmengia honbaensis
